# Monthly Summary

**Published:** 1885-01

**Authors:** 


### Monthly Summary.

---

A Novel Filling.—Case under care of A. H. Tester, D. M. D., L. D. S., Eng.—The following incident in practice may be of interest to our readers: N. B., æt. 27, called on me some time since in order to have his mouth examined. On examining his teeth, I found them remarkably good, with the exception of a few crown cavities. In the first lower molar on the right side I found what appeared to be a very good crown amalgam filling. I examined it carefully, and to all appearances it was very compact, the edges being neatly trimmed and smooth. The patient expressed regret at not having paid proper care and attention to his teeth, and said he had not been to a dentist before. I replied that I thought he must have made a mistake, as I could show him one very good filling in his mouth which I considered very well done, and likely to preserve his tooth foı some time.

He again assured me I was the first dentist he had ever consulted, and as regards the filling I had found it had occurred in the following manner: Last season on returning from duck shooting, he remembered while dining on the proceeds of his day's sport, to have bitten on a shot; which was forcibly driven into a cavity existing in this tooth, which had been slightly sensitive prior to this.

Finding he could not remove it with a toothpick, he had taken to grinding on it, and as it caused him no pain or trouble had eaten on it ever since. I told him I did not wish to remove it, as it had done such good service, but asked him to take special notice, and let me know from time to time how it was progressing.—*British Journal of Dental Science.*

---

STRANGLED TO DEATH BY FALSE TEETH.—Capt. Bankhead, of Grafton, W. Va., a well known civil engineer, who had charge of the survey of the Grafton and Greenbrier Railroad extension from Phillipi, was strangled to death at that place Tuesday night by a set of false teeth becoming loose in his mouth. The Captain had been in ill health for some time, and it was under the influence of chloroform, which was given him during the performance of a delicate operation by his physicians, that the accident occurred. He was a Virginian and and about 60 years of age.

---

MURIATE OF COCAINE.—The Baltimore Surgeons have been using Cocaine extensively in eye and venereal diseases. The reports are very satisfactory. Dr. Michael of the University of Maryland found it very useful in operating upon buboes and cauterizing venereal sores.

TEETH AND BRAIN.—According to Mr. Spence Bate, an English scientific person, the coming race will have no teeth. He alleges that as man advances in development the teeth are gradually evolved into brain matter. This will continue to take place until the time comes when our teeth will be entirely converted into brain, and we shall be compelled either to change our diet or to depend wholly upon false teeth.

It is comforting to know that our brains grow at the expense of our teeth, for we can thereby reconcile ourselves to the prevailing paucity of teeth among Americans. So frail and incapable of prolonged service are American teeth that our dentists have been able to gain the unequaled professional experience that has made them the best dentists in the world. Of course, the meaning of our failure as a teeth-producing people is that we convert them into brain with exceptional rapidity. The more brains a people possesses the fewer teeth they have, and Mr. Spence Bate, were he to examine a thousand American mouths, would unhesitatingly confess that Americans surpass all other nations in the quantity of their brains.

If the coming man finds himself without teeth—as we are told that he inevitably will—it is impossible that he will long adhere to the custom of wearing false teeth. Rather will he frankly acknowledge that he is a toothless animal, and proceed to make the best of his situation. He can, of course, subsist exclusively upon semi-liquid articles of diet; or, in other words, upon what Mr. Logan, with his fondness for Spanish idioms, calls "spoon victuals." There are many animals which thrive upon this sort of diet, and man can undoubtedly add himself to the number. It is more probable, however, that the coming man will consider the disappearance of his teeth as a hint tc him to abandon the clumsy and tedious practice of eating, especially since, by so doing, he can save a great deal of time which he can devote to productive industry.

Why should not the coming man be nourished by hypodermic injections? In this way he could dispense not only with teeth but with his whole stock of digestive organs. Why should a man trouble himself to keep a stomach, a liver, and a number of other eleborate organs, all of which must be kept in repair, or serious consequences will follow? The cost of repairs to the liver alone, in the case of a man living in a malarial region, is a very heavy item, and in many cases it may safely be said that it costs a man more to keep his stomach in operation than the result is worth. If we can be nourished with hypodermic injections—and there is little doubt that before long this will be entirely feasible—wisdom will dictate the abandonment of our digestive organs. These will in a short time beeome merely rudimentary, or will entirely varnish, and their place in the human body will be occupied by the heart and lungs, which will be so greatly enlarged as to take up the entire space once reserved for other organs.

Thus we see the conversion of teeth into brain will finally result in making man independent of his digestive organs and increasing the size of his heart, his lungs, and his brain. Such a man will be far superior physically to the present style of man. The college student of the future will be able to row twice as fast as at present, and the six-day pedestrians will find it as easy to make twelve hundred miles a week as it now is to make six hundred.—*The Odontographic Journal.*

---

THE SOUTHERN DENTAL ASSOCIATION.—The 17th Annual Meeting will be held in New Orleans, commencing Tuesday, March 31st, 1885, and continuing four days. In view of the great Exposition being such an attraction for visitors from all parts of the Country, it is confidently expected that this meeting will be the largest ever held, and of unusual interest.

